# Enhancing protein disaggregation restores proteasome activity in aged cells

**DOI:** 10.18632/aging.100613

**Published:** 2013-11-12

**Authors:** Veronica Andersson, Sarah Hanzén, Beidong Liu, Mikael Molin, Thomas Nyström

**Affiliations:** Department of Chemistry and Molecular Biology, University of Gothenburg, Medicinaregatan 9C, SE-413 90 Göteborg, Sweden

**Keywords:** Replicative aging, yeast, UPS, proteasome, disaggregation

## Abstract

The activity of the ubiquitin-proteasome system, UPS, declines during aging in several multicellular organisms. The reason behind this decline remains elusive. Here, using yeast as a model system, we show that while the level and potential capacity of the 26S proteasome is maintained in replicatively aged cells, the UPS is not functioning properly *in vivo*. As a consequence cytosolic UPS substrates, such as ΔssCPY* are stabilized, accumulate, and form inclusions. By integrating a pGPD-HSP104 recombinant gene into the genome, we were able to constitutively elevate protein disaggregase activity, which diminished the accumulation of protein inclusions during aging. Remarkably, this elevated disaggregation restored degradation of a 26S proteasome substrate in aged cells without elevating proteasome levels, demonstrating that age-associated aggregation obstructs UPS function. The data supports the existence of a negative feedback loop that accelerates aging by exacerbating proteostatic decline once misfolded and aggregation-prone proteins reach a critical level.

## INTRODUCTION

levels of damaged proteins increase with the age of different species, including fungi, flies, worms, bats, birds, rodents, and humans [1-6]. Several principal possibilities have been suggested to explain this apparently universal accumulation of damaged/misfolded proteins, including a diminished capacity for protein quality control, which encompasses the removal of damaged and misfolded proteins by the proteasome [4, 7]. Indeed, the function of the 26S proteasome decreases during aging in several human tissues, senescent primary cultures, and whole organisms [6-10], pinpointing the proteasome as a possible malefactor behind age-related damage propagation.

The 26S proteasome is the essential ATP-dependent protease of the ubiquitin-dependent system (UPS) that serves as a key component in the cellular defence against proteotoxicity by virtue of selectively degrading misfolded and damaged proteins in the cytosol and nucleus. To wit, mutations in the UPS can give rise to neurodegenerative disorders in both rodents and humans [11, 12] and sensitize organisms towards aggregation-prone model proteins of inherited neurological diseases [13, 14]. Moreover, the UPS has recently received special attention in aging research not only because a decline in UPS activity may explain why aging is a risk factor for neurodegenerative diseases [15] but also since elevated UPS activity can extend life span. For example, genetically elevating proteasome levels/activity in the yeast *S. cerevisiae* is extending mother cell life span [16]. In addition, it was recently demonstrated that germline ablation in *C. elegans* extends life span by elevating somatic UPS levels/activity and that ectopic up-regulation of the UPS, through elevated *rpn-6* expression, is sufficient for life span extension [17]. That a trade-off in resource allocation between the germline and soma encompasses the UPS is supported also by data showing that mating of *D. melanogaster* flies has an immediate attenuating effect on the UPS activity of the somatic tissues - specifically, as much as 25% of the somatic UPS capacity of female flies was lost as a result of mating and this was accompanied by an accelerated aging [18]. In this work, we approached the questions of (i) why protein aggregates accumulate in aging yeast mother cells, (ii) if the activity of the UPS in yeast, like animals, is declining with age, and if so, (iii) whether this decline is due to aggregated proteins interfering with UPS performance. We reasoned that *S. cerevisiae* might be an excellent model system to approach these questions as yeasts harbors a unique and efficient protein disaggregase activity - provided by the heat shock protein Hsp104 [19]. The Hsp104 disaggregase, in contrast to the conserved and canonical Hsp70/40 chaperones, cannot prevent protein aggregation and is only affecting protein aggregation by promoting disaggregation [19]. Thus, by creating a yeast strain in which Hsp104 levels are constitutively and stably elevated by integrating a *GPD* promoter-*HSP104* recombinant into the genome, we could address the questions of whether aggregates formed during aging impede UPS function. We report that the *in vivo* UPS activity does indeed decline upon mother cell aging, that elevating UPS levels counteracts age-related aggregate formation, and that constitutively elevating disaggregase activity counteracts age-induced protein aggregation and rescues UPS activity. We discuss the data in the light of proteostasis feedback catastrophe theories of aging.

## RESULTS

### A UPS substrate accumulates and aggregates upon yeast replicative aging

**Figure 1:**
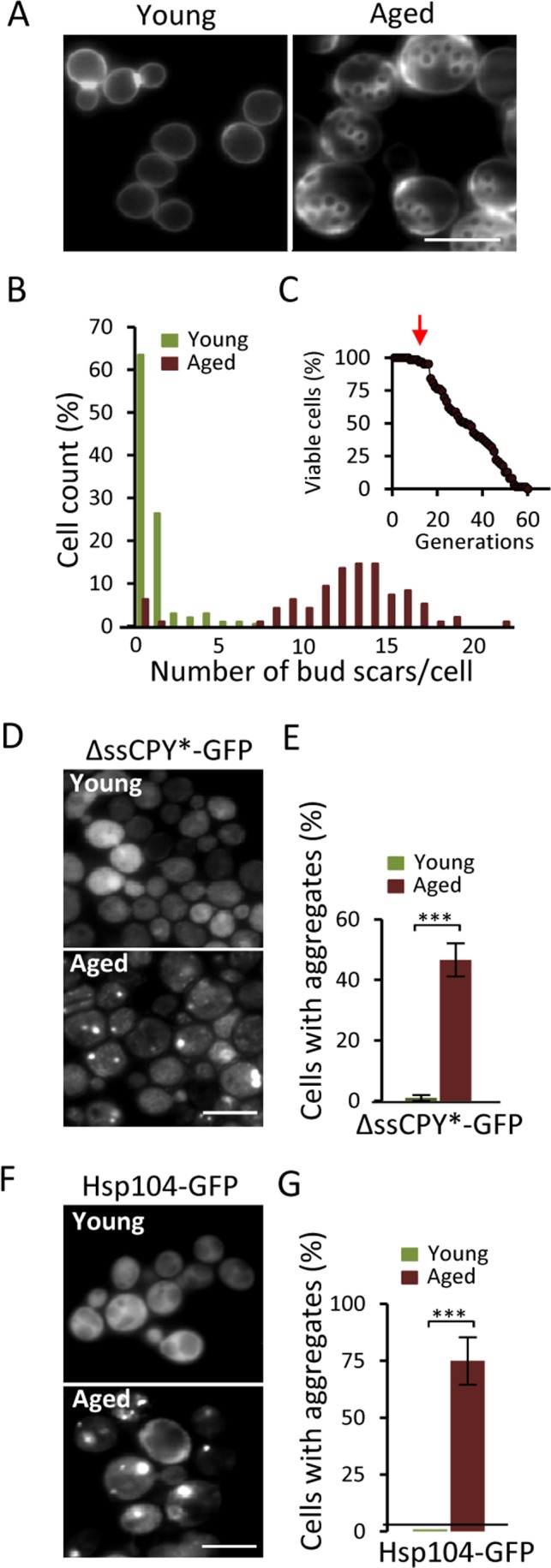
Aged yeast cells accumulate aggregates of the cytosolic UPS substrate ΔssCPY*. (**A**) Representative image of bud scars in young and aged fractions. (**B)** Age distribution in the young and aged mother cell fractions collected. (**C**) The average age (arrow) of the isolated mother cells in relation to the life-span survival curve. (**D**) Localization of ΔssCPY*-GFP in young and aged cells. (**E**) Percentage of cells with ΔssCPY*-GFP foci in young and aged fractions (*n*=3). (**F**) Representative image of Hsp104-GFP distribution in young and aged cells (**G**) Percentage of cells with Hsp104-GFP foci in young and aged fractions (*n*>3). Error bars represent standard deviation. For statistical analyses, the paired two-tailed *t*-test was used ***P<0.001. (*n=* sets of analysis; Scale-bars represent 10μm).

The ΔssCPY*-GFP fusion contains a mutated form of the carboxypeptidase YscY that misfolds in the cytoplasm but is rapidly degraded by the UPS [20]. We used this protein construct to test if aging of yeast mother cells might lead to defects in UPS functions, which would result in the accumulation of misfolded ΔssCPY* and its aggregation. Streptavidin-biotin magnetic sorting (see Materials and Methods) was used to obtain replicatively aged cells of an average age of 13-15 generations (Fig 1A&B). These cells have not yet started to die off but show signs of aging (Fig 1C). As shown in figures 1D&E, there is a dramatic increase in ΔssCPY*-GFP aggregate formation in these aged cells: While no aggregates could be detected in young cells, 50% of the aged cells harbored ΔssCPY*-GFP foci (Fig. 1E). Using Hsp104-GFP, the protein disaggregase acting together with Hsp40/70 [19], as a reporter for protein aggregates [19, 21] confirmed that these aged cells suffer from extensive aggregate formation also of indigenous proteins (Fig 1F&G).

### Proteasome activity is required for aggregate prevention and clearance

**Figure 2:**
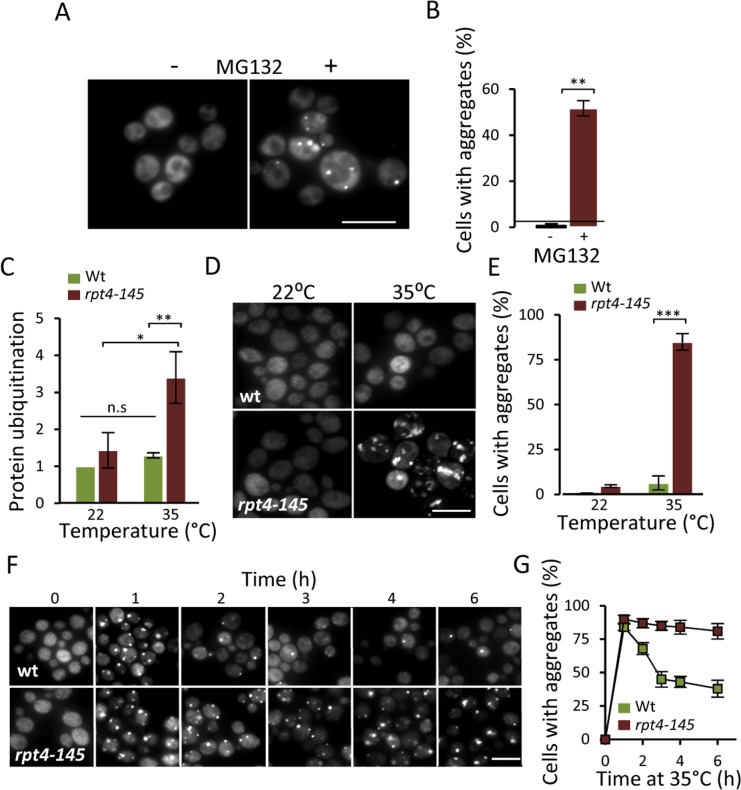
Lowering proteasome function results in increased protein aggregation. (**A**) Representative images of Hsp104-GFP distribution with and without the addition of the proteasome inhibitor MG132 (100 μM) to a growing culture. (**B**) Percentage of cells with Hsp104-GFP foci after partial proteasomal inhibition by MG132. (**C**) Relative levels of protein ubiquitination upon growing the conditional proteasomal mutant *rpt4-145* (ts) at the permissive (22°C) and near non-permissive (35°C) temperature. Levels were normalized to the levels in wt cells grown at 22°C. (**D**) Representative images of Hsp104-GFP localization upon growth of wt and *rpt4-145* cell at the permissive (22°C) and near non-permissive (35°C) temperature. (**E**) Percentage of wt and *rpt4-145* cells with Hsp104-GFP foci after growth at the permissive (22°C) and near non-permissive (35°C) temperature. (**F**) The clearance of Hsp104-GFP foci was followed over time after an initial burst in aggregate formation after the temperature shift. Time point “0” depicts cells growing at 22°C and subsequent time points depict cells following the indicated time at 35°C. (**G**) Percentage of wt and *rpt4-145* cells with Hsp104-GFP foci. Quantification of Hsp104-GFP foci formation in the experiment in “F”. Error bars represent standard deviation (*n=*2). For statistical analysis, the paired two-tailed *t*-test was used where *P<0.05, **P<0.01, ***P<0.001 and *n.s =* no significant difference. (*n=* sets of analysis; Scale-bars represent 10μm).

The results obtained (Fig. 1) raised the possibility that the accumulation of aggregates in aged cells is caused by a failure of the UPS system to properly clear-out misfolded proteins. To approach this possibility, we first tested if there is an increase in Hsp104-GFP foci when artificially lowering the proteasomal activity in a growing culture of predominantly young cells. Indeed, addition of the proteasomal inhibitor MG132, resulted in the immediate accumulation of Hsp104-GFP associated aggregates (Fig 2A&B). In addition to restricting proteasome function chemically, we used the temperature (ts) strain *rpt4-145*; Rpt4 is one of six ATPases of the 19S regulatory particle necessary for proper UPS function [22]. When growing the *rpt4-145* strain at a near non-permissive temperature (35°C), the level of ubiquitinated proteins increased 3-fold compared to wt and growth at the permissive temperature (22°C) (Fig 2C) confirming that *rpt4-145* indeed has a defect in proteasome function at the temperature tested. Moreover, Hsp104-GFP foci accumulated when the cells were grown at 35°C (Fig 2D&E) demonstrating that an unperturbed proteasome activity is necessary to prevent aggregate formation in young cells. We next tested if functional proteasomes are required also for the clearance of the aggregates once formed. To accomplish this we induced aggregate formation by switching the growth temperature from 22°C to 35°C and monitored aggregate clearance over time. After an initial surge in aggregate formation, which occurred to a similar extent in both wt and *rpt4-145* cells upon the heat stress, aggregates formed in the wt strain were resolved at a markedly higher rate (Fig 2F&G). The data indicate that proteasome activity is necessary both for preventing aggregate formation in young cells and for the efficient clearance of stress-induced protein aggregates.

### Boosting proteasome production mitigates aggregate formation in aged cells

**Figure 3:**
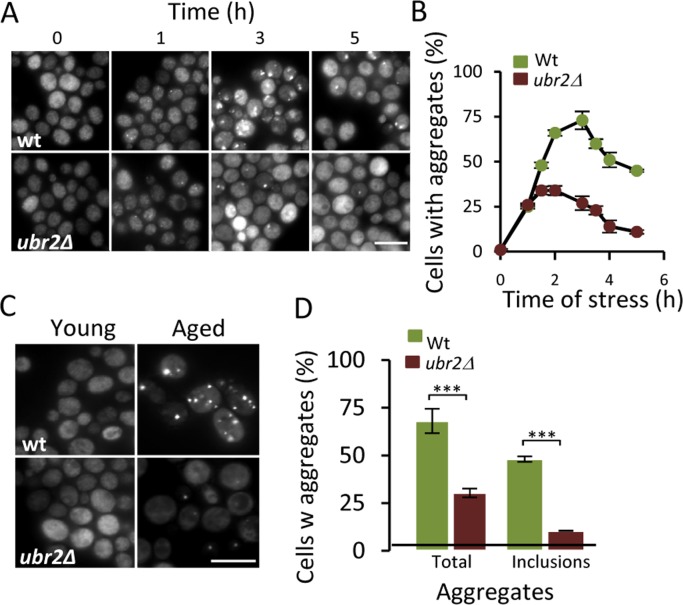
Increasing proteasome production reduces aggregate formation. (**A**) Representative images of Hsp104-GFP distribution in wt and *ubr2Δ* cells after H_2_O_2_ exposure (0.6 mM). Time point “0” depicts cells before stress whereas subsequent time points depict cells at the indicated time point following the addition of H_2_O_2._ (**B**) Percentage of wt and *ubr2Δ* cells with Hsp104-GFP foci over time after peroxide stress (*n=*2). (**C**) Representative image of Hsp104-GFP localization in young and aged (~13-15 generations) wt and *ubr2Δ* cells. (**D**) Percentage of aged wt and *ubr2Δ* cells containing Hsp104-GFP foci (*n≥*3). “Total” represents the percentage of cells containing foci independent of size, whereas “inclusions” represent the percentage of cells with large foci. Error bars represent standard deviation. For statistical analysis, the paired two-tailed *t*-test was used where *P<0.05, **P<0.01, ***P<0.001 and *n.s =* no significant difference. (*n=* sets of analysis; Scale-bars represent 10μm).

To further test if there is a link between the UPS and protein aggregation, we tested the effect of increasing proteasome activity on Hsp104-GFP foci formation by deleting *UBR2*. Loss of the E3 ubiquitin ligase Ubr2 stabilizes the transcription factor Rpn4, which is a positive regulator of genes encoding proteasomal subunits [23] - increased Rpn4 levels leads to more proteasomes and elevated proteasome capacity [16]. To determine if the proteasome is a bottleneck for aggregate management, we induced aggregates by hydrogen peroxide exposure (0.6 mM) and found that the *ubr2* mutant displayed 50% lower levels of Hsp104-GFP foci compared to wt (Fig 3A&B) indicating that elevating proteasome capacity helps in preventing aggregate formation. We wondered whether age-induced aggregation was similarly affected by boosting proteasome production and found that aged cells lacking Ubr2 displayed markedly less Hsp104-GFP foci and large Hsp104 associated inclusions (Fig 3C&D). These data suggest that the UPS may be limiting for proper protein quality control during cellular aging leading to aggregate formation.

### Overproducing Hsp104p decreases protein aggregation and restores diminished proteasome function in aged cells

**Figure 4:**
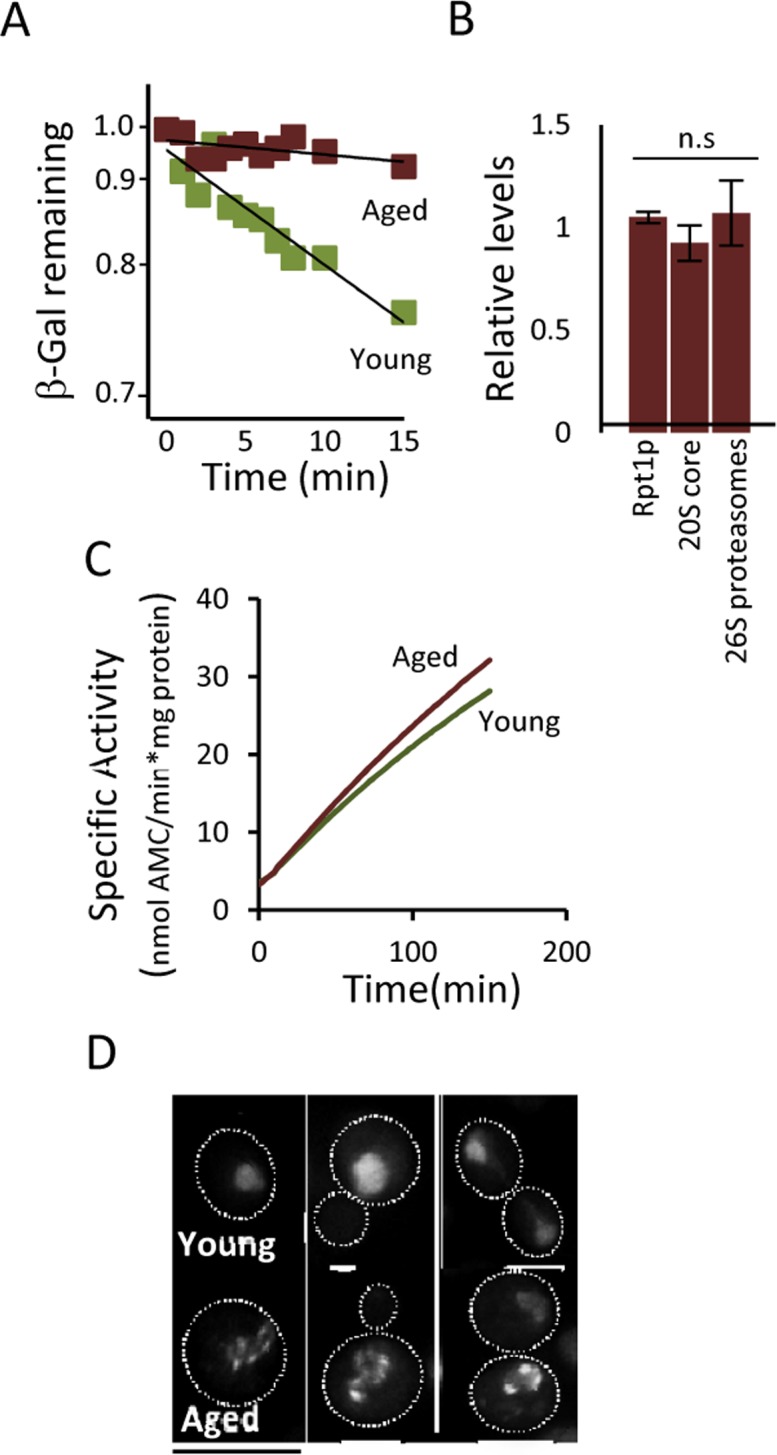
Proteasome function is diminished in aged cells. (**A**) Degradation of the *in vivo* UPS substrate ub-P-βgal over time in young and aged (~13-15 generations) cells after the inhibition of protein synthesis. The starting β-gal levels were set to 1. The figure depicts representative results from one out of six independent experiments (*P*=7.38^E-06^). (**B**) Relative levels of Rpt1p (19S subunit), 20S core proteins, and 26S proteasomes (based on native gels) in aged cells compared to young cells (*n≥*3). (**C**) Proteasomal capacity in total protein extracts measured as the rate of hydrolysis of the fluorogenic peptide suc-LLVY-AMC (Chymotryptic activity) depicted as the specific activity (nmol AMC/min*mg total protein). A representative figure is presented (*n=*3). (D) Rpt6-GFP (19S subunit) localization in young and aged (~13-15 generations) cells. Error bars represent standard deviation. For statistical analysis, the paired two-tailed *t*-test was used where *P<0.05, **P<0.01, ***P<0.001 and *n.s =* no significant difference. (*n=* sets of analysis; Scale-bars represent 10μm.

**Figure 5:**
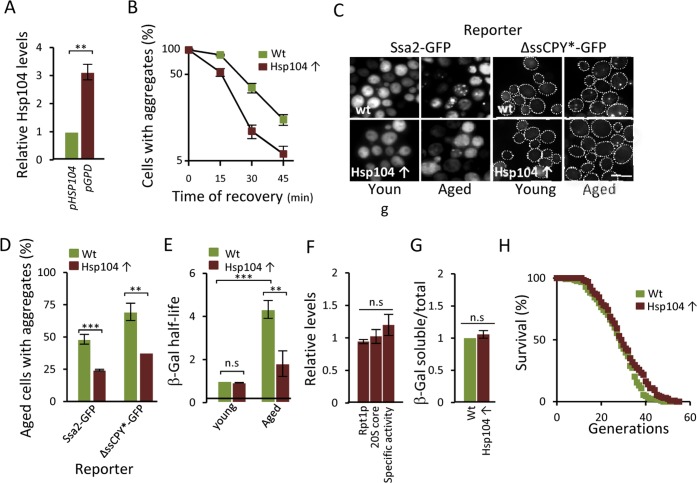
Overproducing Hsp104 mitigates aggregate accumulation and restores proteasome function in aged cells. (**A**) Relative levels of Hsp104 produced from the wt *HSP104* and *GPD* promoters as determined by anti-Hsp104 immuno-blot analysis (*n=*2). (**B**) Percentage of cells with Ssa2-GFP foci following heat stress in the wt and Hsp104 overproducing (Hsp104↑) strains. Time point “0” represents cells after 30 min incubation at 42°C, whereas subsequent time points represent cells following the indicated time of recovery at 30°C (*n=*2). (**C**) The effect of Hsp104 overproduction on aggregate formation. Representative image of Ssa2-GFP and ΔssCPY*-GFP in young and aged, wt and Hsp104 overproducing cells. (**D**) Percentage of aged wt and Hsp104 overproduction cells with Ssa2-GFP or ΔssCPY*-GFP foci (*n=*2). (**E**) Relative half-life of β-gal in wt and Hsp104 overproducing young and aged cells. Values were normalized to the half-life in wt young cells (*n*=3). (F) Relative levels of Rpt1p (19S subunit), 20S core proteins, and proteasome specific activity (rate of hydrolysis of suc-LLVY-AMC) in Hsp104 overproducing cells compared to wt cells. (*n≥*2). (**G**) Relative levels of soluble β-gal in wt and Hsp104 overproducing cells normalized to total protein (see Experimental procedures for details) (*n=*2). Error bars represent standard deviation. For statistical analysis, the paired two-tailed *t*-test was used where *P<0.05, **P<0.01, ***P<0.001 and *n.s =* no significant difference. (Scale-bar represents 10μM). (H) Life-span curves of wt and Hsp104 overproducing cells. Mean replicative life-spans are: wt (28 ± 0), Hsp104 overproduction (29.5 ± 1.5) (*n=*2). (*n=* sets of analysis).

The fact that (i) a modest reduction, chemically or genetically, of proteasome activity leads to aggregate formation already in young cells, that (ii) boosting proteasome levels/activity diminishes aggregate formation in aged cells, and that (iii) a substrate (ΔssCPY*) for the proteasome accumulates as aggregates during aging, suggest that UPS is a key factor in protein homeostasis that becomes limiting in aged yeast cells. To test this more directly, a ubiquitinated β-galactosidase (ub-Pro-βgal) was used as an *in vivo* substrate for the 26S proteasome. We found that while this substrate was efficiently degraded in young cells, aged cells failed to do so (Fig 4A). This failure of aged cells to degrade ub-Pro-βgal was not associated with an apparent diminished abundance of proteasomes, as shown in Fig 4B. Moreover, the proteasome capacity of aged cells was not reduced, as analyzed by the processing of Suc-LLVY-AMC in protein extracts (Fig 4C). Thus, proteasomes do not appear to be non-functional and terminally damaged in aged cells. Nevertheless, it appears that the proteasomes are prevented from functioning properly *in vivo*, possibly by insoluble/aggregated proteins interfering with proteasome functions. In support of this, we found that the localization of a proteasome subunit (Rpt6-GFP) was redistributed from being predominantly nuclear in young cells to become increasingly associated with extra-nuclear granular structures during aging (Fig. 4D). To test the hypothesis that aggregates obstruct UPS function in aged cells, we incorporated a genomic *HSP104* controlled by the strong *GPD* promoter to elevate disaggregase activity. This recombinant construct displayed an elevated level of Hsp104 (Fig 5A) and a more rapid clearance of stress-induced aggregates as analyzed using the chaperone Hsp70, Ssa2-GFP, as a reporter (Fig 5B). We then tested if elevated Hsp104 levels resulted in altered protein aggregation in aged cells. Indeed, the accumulation of Hsp70-associated aggregates and ΔssCPY*-GFP inclusions upon aging were decreased by overproducing Hsp104 (Fig 5C-D).

Remarkably, overproduction of Hsp104 also markedly reduced the half-life of ub-Pro-βgal in aged cells (Fig 5E) in the absence of an increase in the levels of proteasomal subunits and capacity (Fig 5F). To test if the increased ub-Pro-βgal degradation in response to elevated Hsp104 levels was due to the extraction of the ub-Pro-βgal substrate itself from aggregates, we quantified the amount of soluble and aggregated forms of ub-Pro-βgal in both young and aged cells. We found that the levels of soluble β-gal compared to total amount, was similar in both the wt and Hsp104 overproducing strain (Fig 5G) suggesting that soluble substrate was not limiting in aged cells. Taken together, the data suggest that misfolded proteins accumulate upon aging of yeast mother cells, in part, as a result of a diminishing functionality of the proteasomes present, which in turn appears to be a consequence of insoluble, aggregated, proteins interfering with the normal task of the UPS. However, the increased proteasome activity achieved by Hsp104 overproduction did not significantly increase life span (Fig 5H) indicating that the proteasome activity is not elevated to levels high enough to extend life-span e.g. to those reached by deleting *UBR2* [16]. In support of this, we failed to determine the half-life of ub-Pro-βgal in both young and aged *ubr2Δ* cells due to the extraordinarily rapid degradation of B-gal.

## DISCUSSION

Previous reports have shown that accumulation of protein aggregates of model disease proteins of human neurological disorders impairs the functional capacity of the UPS [24-27] but to what extent aggregation of indigenous proteins is causing a decline in UPS performance during aging has not been shown. Herein, we report evidence for that UPS activity is declining during replicative aging of yeast mother cells and that this decline is intimately linked to protein aggregation interfering with UPS performance. Specifically, we show that elevated disaggregate activity restores UPS-dependent degradation *in vivo* in aged cells without elevating UPS levels.

There are a number of possible mechanisms by which increased disaggregation may restore UPS functions. For example, subunits of the proteasome might themselves aggregate during aging leading to a decreased abundance of fully assembled and active 26S proteasomes. However, aging did not result in a diminished abundance of the 26S proteasome or a reduced proteasomal capacity as judged by the efficient degradation of the fluorogenic substrate Suc-LLVY-AMC in protein extracts. It has been suggested that aggregated poly-glutamine [28, 29] and oxidatively damaged [30] proteins are degradation-resistant and the engagement of the UPS with such aggregates will inhibit (‘clog’ or ‘choke’) UPS function *in vivo*. In this scenario, increased Hsp104 disaggregation would cause a de-clogging of the UPS. It is also possible that factors required for UPS activity, such as ubiquitin ligases/proteases or proteasome activators, are becoming sequestered in aggregates during aging and retrieved from such aggregates by elevated disaggregation. The latter two possibilities are both compatible with a maintained *in vitro* 26S proteasome capacity in extracts of aged cells as the extraction protocol might release proteasome inhibition by releasing aggregate-proteasome engagement and because the ubiquitin/de-ubiquitin systems are not required for Suc-LLVY-AMC processing.

**Figure 6:**
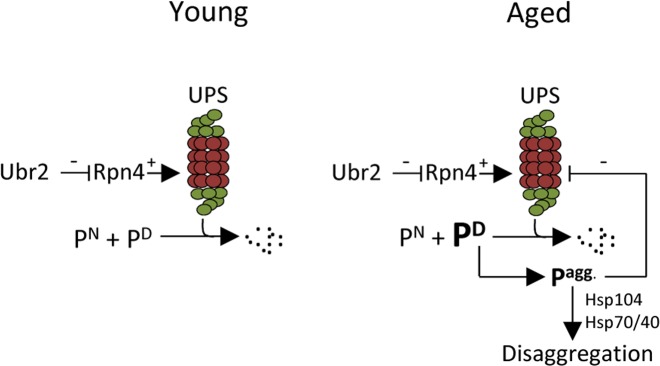
Schematic representation of how aggregated proteins might result in a negative proteostasis feedback loop. One of many cellular functions of the UPS is to degrade native (e.g. cell cycle regulators), damaged, or aberrant proteins. If the level of damaged proteins exceeds the proteasomal capacity or if UPS degradation is somehow compromised, protein aggregates will form. The disaggregase Hsp104 can together with Hsp70/40 resolve protein aggregates. However, if the accumulation of aggregated protein is too severe, as seen in aged cells, these may interfere with the proper function of the UPS creating a negative feedback loop. This study indicates that the buildup of aggregates in aged cells can be counteracted either by increasing the amount of proteasomes present by stabilizing Rpn4, through the deletion of *UBR2*, or by increasing the level of disaggregation through Hsp104 overproduction. [UPS=ubiquitin proteasome system; P^N^= native protein; P^D^= damaged protein P^agg.^= aggregated proteins].

It has been shown previously that a *ubr2Δ* mutant, which displays elevated UPS levels and activity, displays a robust extension in replicative life span [16]. In addition, aggregation of the poly-glutamine protein Htt103Q is ameliorated in young, unsorted, *ubr2Δ* cells [16]. In this work, we expanded on these observations and show that elevated UPS activity also diminishes aggregation of indigenous proteins during aging. Moreover, a close link between aggregate formation/disaggregation and proteasome activity is evidenced by the fact that aggregates accumulated to a much lesser extent upon peroxide stress in *ubr2Δ* mutants and that *rpt4* mutants displayed a retarded disaggregation. The intimate interdependence of the UPS and disaggregation machineries may lead to a negative feedback loop once the levels of misfolded proteins, or any aggregation-prone substrate of the UPS, reach levels exceeding the capacity of the UPS (Fig. 6). The aggregation of such proteins is negatively affecting UPS performance as discussed above, which, in turn, leads to an accelerated accumulation and formation of aggregated UPS substrates (Fig. 6). Since the UPS regulates a large variety of cellular processes, including the cell cycle through regulated destruction of transcriptions factors, cyclins, and protein kinases [31-33], an aggregation-dependent inhibition of UPS performance is most likely leading to aberrant cell cycle progression; a hallmark of aging cells. We show herein two ways to counteract such an accumulation of aggregates formed upon aging; (i) by boosting the levels of active 26S proteasomes, and (ii) by elevating protein disaggregase activity (Fig. 6).

We realize that the negative feedback loop described cannot explain, without using circular arguments, the onset of aging and why misfolded proteins accumulate in the first place but might explain how aging is accelerated once critical levels of protein damage are reached. It has recently been shown that a collapse in vacuole acidification and performance is an early event in yeast aging, which drives the subsequent dysfunction of mitochondria [34]. It is thus plausible that vacuolar dysfunctions in the mother cells lead to ramifications in cytosolic/nuclear protein homeostasis leading to elevated levels of damaged proteins. In addition, asymmetrical inheritance of both damaged and functional proteins encompasses proteins involved in ROS defences [21] resulting in elevated levels of peroxides in mother cells [21, 35], which might lead to damage and aggregation of UPS substrates. The role of peroxides in replicative aging of yeast mother cells was recently evidenced by the fact that boosting peroxiredoxin activity extends replicative life span [36] but it is presently unknown if this life span extension is linked to the prevention of protein damage, aggregation, and UPS performance.

More than a decade ago it was reported that fibroblasts of centenarians display an elevated UPS capacity [37], a feature shared by the long-lived organism the naked mole rat [38]. Recent reports demonstrating that boosting UPS activity can prolong life span provide evidence for a more direct causal relationship between longevity and the UPS [16, 17]. Such data, together with data reporter herein, highlights that protein quality control may be a bottleneck in longevity assurance and that modulations in protein homeostasis greatly contribute to life span control. To what extent protein homeostasis, including the UPS and disaggregation machinery, can be targeted in therapeutic gerontology to retard or postpone the onset of age-related neuro-degeneration appears to be a question well worth pursuing.

## METHODS

### Yeast strains and growth conditions

The *S. cerevisiae* strains used in this study are all derived from BY4741 (*MATa; his3Δ1; leu2Δ0; met15Δ0; ura3Δ0*). C-terminally GFP-tagged *HSP104, SSA2, PRE5* and *RPT6* strains were from a yeast GFP collection (Invitrogen) [39]. To overexpress *HSP104*, pGPD-*HSP104* was amplified from plasmid pYM-N15 [40] using the following primers: (s1) 5' - CCT TTT TAC CCT TGA ATC GAA TCA GCA ATA ACA AAG AAA AAA GAA ATC AAC TAC ACG TAC CAT AAA ATA TAC AGA ATA TAT GCG TAC GCT GCA GGT CGA C - 3' and (s4) 5' - GGA TGT TGA TGA TCC GAA GCC AAT TTT TGA GCC AAC GTC AAA ATC GTT AGA GCC CTT TCT GTA AAT TGC GTT TGG TCG TTC ATC GAT GAA TTC TCT GTC G - 3'. The ub-P-βGAL and control ub-M-βGAL (pUB23-X-beta-gal) plasmids used were first described in [41] and were transformed into the desired strains. The plasmid pRS316-ΔssCPY*-GFP is described in [42] and was transformed into the desired strains (the plasmid was a kind gift from Prof. Dieter H. Wolf). The Hsp104-GFP conditional proteasome mutant *rpt4-145* (*rpt4-145::kanMX4)* and the *ubr2Δ* mutant (*ubr2Δ::kanMX4)* strains were constructed by Synthetic Genetic Array method where *his3Δ* Hsp104-GFP (*MAT*a *his3Δ::kanMX4 can1Δ::STE2pr-Sp_his5 lyp1Δ ura3Δ0 leu2Δ0 met15Δ0 LYS2+ HSP104::HSP104-GFP-LEU2)* was used as wt.

Unless otherwise stated, cells were grown at 30°C in rich (YPD) medium containing 2% peptone, 1% yeast extract and 2% glucose or in synthetic defined (SD) medium containing complete supplement mixture (CSM) with or without the indicated amino-acids, 0,17% yeast nitrogen base (pH 5.5), 0.5% ammonium sulfate and 2% glucose to mid-exponential phase.

### Isolation of old cells

Old cells were obtained by magnetic sorting [43] with modifications. In short, cells were harvested at mid-exponential phase and labeled with 1mg/ml of EZ-Link Sulfo-NHS-LC Biotin (Thermo Scientific, Rockford, IL, USA). Any access of biotin was washed away and after an overnight growth, the cells were incubated at 4°C for 2 hours with 2,5×10^7^ streptavidin-conjugated magnetic beads/ml. Labeled cells were magnetically sorted in the presence of glucose (or galactose where needed) and then grown overnight. Cells were once again incubated with streptavidin-coated magnetic beads and unlabelled cells were washed away. The sorting efficiency was assessed by Calcofluor White (Sigma-Aldrich, Stockholm, Sweden) staining and bud scar counting. For microscopy, cells were fixed in 3.7% formaldehyde and washed three times in PBS.

### *In vivo* proteasome activity

2μ-based plasmids carrying the ubiquitin-*lacZ* gene fusion Ub-Pro-βgal and the stable control ub-Met-βgal [41] were transformed into the desired strains. Cells were grown in SD-ura with 2% galactose at 30°C. Young and aged cells were isolated as described above (Smeal, Claus et al. 1996), resuspended in SD-ura 2% Gal and cycloheximide was added to a final concentration of 0.5 mg/ml. Samples for β-gal measurements were taken before as well as repeatedly after the addition of cycloheximide.

### Protein extraction and concentration

Harvested and washed cells were resuspended in lysis buffer A (15% Glycerol; 50 mM Tris-HCl, pH 7.5; 2 mM ATP; 5 mM MgCl_2_; 1 mM DTT; +/− protease inhibitor cocktail (Roche, Bromma, Sweden)) or lysis buffer B (10% glycerol; 25mM tris-HCl, pH 7.5; 10 mM MgCl_2_; 4 mM ATP, pH7.5; 1mM DTT) and lysed with 0.5mm glass beads using a FastPrep-24 instrument (MP Biomedicals, Illkirch Cedex, France). Cells were vortexed 5 times at 4°C for 20 seconds at speed 6.0 m/s with a 2 min recovery on ice between each vortex. Samples were briefly centrifuged to sediment cell debris and protein concentration was determined using the D_C_ Protein Assay (Bio-Rad, Sundbyberg, Sweden). Protein standard was made using 2 mg/ml bovine serum albumin (Sigma-Aldrich).

### *In vitro* proteasome capacity assay

Cell lysates were prepared as described above in lysis buffer A (without protease inhibitor) and the *in vitro* proteasome capacity was measured through the rate of hydrolysis of the fluorogenic peptide Suc-LLVY-AMC (succinyl-Leu-Leu-Val-Tyr-7-amino-4-methylcoumarin; Bachem, Saffron Walden, U.K.) representing the proteasomal chemotryptic activity. In 96-well plate, 20 μg of protein from each extract was incubated at 30°C with 200μM suc-LLVY-AMC in 25 mM Tris-HCl, pH 7.5; 5 mM ATP, pH 7.5 for assay of 26S activity in a total volume of 200 μl. AMC fluorescence was measured with the Synergy™ 2 microplate reader (BioTek^®^, Winooski, VT, USA) using 390-nm excitation and 460-nm emission filters and as a standard, free AMC (Bachem) was used. 20 μM of the proteasomal inhibitor MG132 (PI102; Enzo Life Sciences, Solna, Sweden) was added to some wells as a control for proteasome activity. This concentration of MG132 inhibited activity by approx. 90%.

### Gel electrophoresis and immunoblotting

For protein separation by SDS-PAGE samples were prepared using a modified Laemmli sample buffer (3% SDS; 2.5% β-mercaptoEtOH; 0.001% bromphenol blue; 30 mM Tris-HCL pH 6.8) or standard SDS-PAGE protocol. Proteins were separated using precast NuPAGE 10% Acrylamide Bis-Tris gels (Invitrogen, Stockholm, Sweden). For native-PAGE, cells were lysed in buffer B as described above and loaded onto native gels containing 4% acrylamide in Tris/borate buffer optimized for preservation of proteasomal complexes. Gels were blotted using the Hoefer (Holliston, MA, USA) system at 40V, 125mM, ON onto PVDF (poly vinylidene difluoride) membrane (Millipore, Solna, Sweden) using a 20% Methanol transfer buffer. Immunodetection was carried out using the following primary antibodies: anti-rpt1 (PW8255; Enzo life sciences); anti-core (PW9355, Enzo life sciences); anti-hsp104 (ab69549; Abcam, Cambridge, UK); anti-polyubiqiutin (ab7254; Abcam); anti-βgal according to manufacturer's recommenda-tions. As secondary antibodies, goat anti-Mouse IRDye 680 or 800CW and goat anti-Rabbit IRDye 680 or 800CW (LI-COR) were used. Membranes were scanned using the LI-COR Odyssey Infrared scanner. Finally the membranes were stained briefly with coomassie, destained over-night and used as a protein loading control together with a non-blotted coomassie stained gel scanned using the Odyssey scanner.

### Solubility Assay

Solubility assay was carried out as described [44] with some minor adjustments. Briefly, 50 ml exponentially growing culture/sample was washed with four volumes of 20 mM Sodium Azide before resuspended in 1 ml Ice-cold Sorbitol Lysis buffer (0.7 M Sorbitol; 50 mM Tris-HCl, pH 7.5; protease inhibitor cocktail (Roche)) and lysed using glass beads (see above). Samples were centrifuged at 4°C, 300xg for 5 min. The samples were divided into two fractions of 400 μl each, where one fraction (soluble protein; aggregated protein) was subjected to ultracentrifugation at 4°C, 130,000xg for 30 min, while the other fraction (total protein) was kept on ice. After ultracentrifugation, the pellet was washed with sorbitol lysis buffer and ultracentrifuged again while the protein in the supernatant, as well as the total protein fraction were TCA precipitated (11%), washed with 80% acetone and left to dry. All protein fractions were solubilized with 60 μl Urea buffer (40 mM Tris-HCl, pH 6.8; 8 M Urea; 5% SDS; 100 mM EDTA, pH 8.0; 1.5% β-mercaptoethanol; Bromophenol Blue (200 μg/ml)) and boiled for 15 min. Protein content determination and separation was performed as described above.

### Inhibition of proteasome activity

Cells were grown to mid-exponential phase as described above and treated for one hour with either DMSO (control) or 100 μM MG132 (Enzo Life Science). For microscopy, cells were fixed in 3.7% formaldehyde and washed three times in PBS.

Wt and *Rpt4-145* (ts-strain) harboring the HSP104-GFP reporter were grown in YPD 2% glucose at permissive temperature (22°C) and near non-permissive temperature (35°C) until mid-exponential phase and either fixed in 3.7% formaldehyde and washed three times in PBS for microscopy or washed in lysis buffer A as described above and stored at -80°C for western analysis.

For the aggregation clearance experiment, wt and *rpt4-145* harboring the HSP104-GFP reporter in the SGA background were first grown in YPD 2% glucose at permissive temperature (22°C) until mid-exponential phase and then transferred to the near non-permissive temperature (35°C). Growth was monitored over time and samples taken for microscopy as described.

### Hydrogen peroxide and heat stress

Wt and *ubr2Δ* harboring the HSP104-GFP reporter in the SGA background were grown in YPD 2% glucose to mid-exponential phase and treated with 0.6 mM H_2_O_2_ and samples were taken over time. For microscopy, cells were fixed in 3.7% formaldehyde and washed three times in PBS.

Exponentially growing wt and OE *HSP104* strains harboring the Ssa2-GFP were incubated at 42°C for 30 min, before being left to recover at 30°C. Samples were taken continuously and fixed for microscopy as described above.

### Life-span analysis

Life-span analysis was carried out as previously described [21, 45]. Briefly, virgin cells from exponentially growing cultures were placed on YPD plates using MSM Singer Micromanipulator (Singer Instruments, Roadwater, Somerset, UK). The daughter cells from each mother were removed and the number of daughters produced by each mother was recorded. The median life-span was calculated from the number of generations where 50% of the mother cells were producing daughters.
